# Radiomics of spinal muscles: toward a radiological biomarker for allograft rejection in lung transplant

**DOI:** 10.1007/s11547-023-01674-x

**Published:** 2023-07-17

**Authors:** Chiara Giraudo, Antonella Modugno, Giacomo Negro, Andrea Dell’Amore, Diego Cecchin, Raffaella Motta, Elisabetta Balestro, Annalisa Boscolo, Fiorella Calabrese, Eleonora Faccioli, Paolo Navalesi, Andrea Vianello, Federico Rea, Roberto Stramare

**Affiliations:** 1 Unit of Advanced Clinical and Translational Imaging, Department of Medicine – DIMED, University of Padova, Padua, Italy; 2 Thoracic Surgery Unit, Department of Cardiac, Thoracic, Vascular Sciences and Public Health, University of Padova, Padua, Italy; 3 Nuclear Medicine Unit, Department of Medicine – DIMED, University of Padova, Padua, Italy; 4 Respiratory Disease Unit, Department of Cardiac, Thoracic, Vascular Sciences and Public Health, University of Padova, Padua, Italy; 5 Anesthesiology and Intensive Care Unit, Department of Medicine-DIMED, University of Padova, Padua, Italy; 6 Pathological Anatomy Section, Department of Cardiac, Thoracic, Vascular Sciences and Public Health, University of Padova, Padua, Italy; 7 Respiratory Pathophysiology Division, Department of Cardiac, Thoracic, Vascular Sciences and Public Health, University of Padova, Padua, Italy

**Keywords:** Lung transplant, Radiomics, Muscle composition, Computed tomography

## Abstract

**Purpose:**

To assess the role of muscle composition and radiomics in predicting allograft rejection in lung transplant.

**Material and methods:**

The last available HRCT before surgery of lung transplant candidates referring to our tertiary center from January 2010 to February 2020 was retrospectively examined. Only scans with B30 kernel reconstructions and 1 mm slice thickness were included. One radiologist segmented the spinal muscles of each patient at the level of the 11th dorsal vertebra by an open-source software. The same software was used to extract Hu values and 72 radiomic features of first and second order. Factor analysis was applied to select highly correlating features and then their prognostic value for allograft rejection was investigated by logistic regression analysis (level of significance *p* < 0.05). In case of significant results, the diagnostic value of the model was computed by ROC curves.

**Results:**

Overall 200 patients had a HRCT prior to the transplant but only 97 matched the inclusion criteria (29 women; mean age 50.4 ± 13 years old). Twenty-one patients showed allograft rejection. The following features were selected by the factor analysis: *cluster prominence, Imc2, gray level non-uniformity normalized, median, kurtosis, gray level non-uniformity, and inverse variance*. The radiomic-based model including also Hu demonstrated that only the feature *Imc2* acts as a predictor of allograft rejection (*p* = 0.021). The model showed 76.6% accuracy and the *Imc2* value of 0.19 demonstrated 81% sensitivity and 64.5% specificity in predicting lung transplant rejection.

**Conclusion:**

The radiomic feature *Imc2* demonstrated to be a predictor of allograft rejection in lung transplant.

## Introduction

Lung transplantation is a major surgical procedure for patients with end-stage pulmonary diseases such as idiopathic pulmonary fibrosis [1]. Since the first transplant performed by Hardy in 1963 the delivered care in this field has significantly improved in terms of surgical techniques, candidate selection, post-operative immunosuppressive treatment, and diagnosis and management of organ rejection [1, 2]. Regarding this last aspect, allograft rejection is due to an immune response toward the transplanted organ and can be categorized as hyperacute, acute, and chronic. The hyperacute response is usually caused by preformed antibodies of the recipients against the human leukocyte antigen (HLA) of the donor, the acute form can be T-cell or antibody mediated while the chronic type is still considered multifactorial with various risk factors contributing to its occurrence (e.g., infections and HLA-mismatching) [3–5]. The diagnosis of rejection requires a specialized multidisciplinary team including a thoracic radiologist [5–7]. Computed tomography (CT) signs of acute primary graft dysfunction and acute allograft rejection comprise ground-glass opacities, consolidation, interstitial thickening, and pleural effusion while chronic allograft lung dysfunction can be characterized at imaging by bronchiectasis, small nodules, and air trapping as part of the bronchiolitis obliterans syndrome pattern or by reticulation and upper lobes fibrosis representative of the restrictive allograft syndrome [7, 8].

In the complex management of the post-transplant phase, also the impact of muscle composition has been assessed by mechanical tests and imaging [9–12]. Various radiological techniques can be used for muscle assessment including ultrasound, dual-energy X-ray absorptiometry (DXA), CT, and magnetic resonance (MR). For instance, by ultrasound information regarding muscle thickness, architecture and composition such as muscle echogenicity, fiber length, and pennation angle can be collected [13]. By DXA several parameters reflecting body composition and muscle status can be obtained, like the appendicular lean mass and the appendicular lean mass index. Nevertheless, it should not be overlooked that these variables should be associated with a qualitative assessment performed by physical evaluation [14]. CT allows the extraction of quantitative data like muscle density, cross-sectional area, and the skeletal muscle index while MR enables a qualitative and quantitative assessment of muscle structure and characteristics by semi-quantitative scores applied on turbo spin-echo images or using specific sequences such as Dixon, Diffusion Weighted Imaging (DWI), and mapping, respectively [15, 16].

Nowadays, advanced quantitative imaging techniques like radiomics and machine learning have been successfully used also to investigate muscle characteristics showing promising results in predicting sarcopenia in patients with non-small cell lung cancer [17–19]. These methods which are based on complex computational analyses of radiological images providing crucial information beyond the visible were initially applied in the oncological field but as mentioned above are currently used for different types of diseases [20–23].

Nevertheless, until now, to the best of our knowledge, the application of radiomics on muscles in transplantology has not been explored yet.

Thus, aim of this study was to evaluate if muscle composition, expressed as muscle density, and radiomics of the paravertebral muscle act as predictors of allograft dysfunction in bilateral lung transplant candidates.

## Methods

### Study design

The last available high-resolution chest CT (HRCT) before surgery of bilateral lung transplant candidates (i.e., performed in the two months preceding operation) referring to our tertiary center from January 2010 to February 2020 was examined for this retrospective institution review board approved study. The following inclusion criteria were applied: scans performed with volumetric acquisitions, 1 mm slice thickness, and B30 kernel reconstructions.

For each patient, clinical and first-line laboratory variables [body mass index (BMI), Barthel index, creatinine (umol/L), white (× 10^9^ L) and red blood cell (× 10^12^ L) count, hematocrit (vol%), hemoglobin levels (g/dl), and erythrocyte sedimentation rate (ESR) (mm/h)] were collected.

### Muscle segmentation and radiomic analysis

**Fig. 1:**
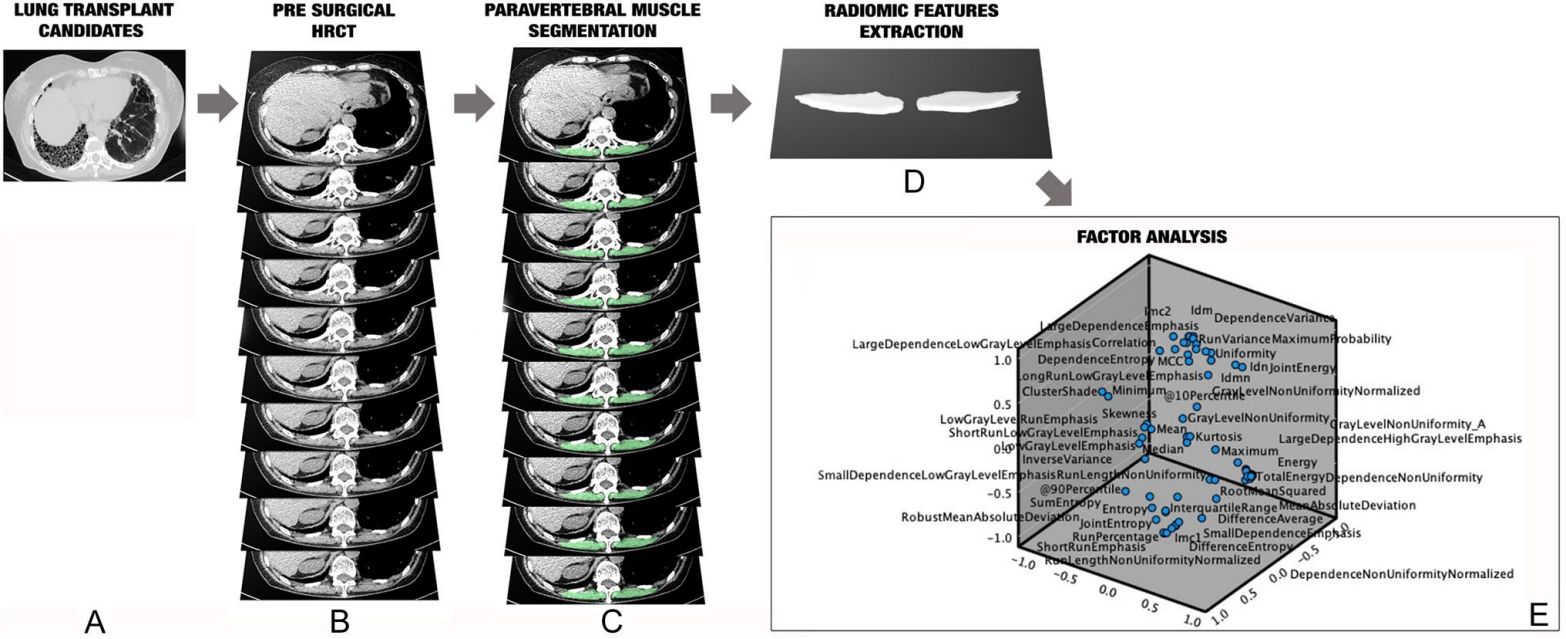
Graphic representation of the study design: in **A** axial HRCT scan indicating the examined population, composed of candidates to bilateral pulmonary transplant referring to our tertiary center; in **B** the selection of the paravertebral muscles at the level of the 11th dorsal vertebra with an extension of 1 cm height, used for the segmentation; in **C** the segmented muscle area, performed applying the threshold − 29 to + 150 Hu; in **D** the examined volume from which were extracted the radiomic features; in **E** the factor analysis applied for variables reduction

One radiologist with ten years of experience in chest and musculoskeletal imaging used a semi-automatic tool of an opens source software (3D Slicer, www.slicer.org) to segment the spinal muscles of each patient at the level of the 11th dorsal vertebra, covering a volume of 1 cm height, and applying the usual Hu range for muscle assessment (− 29 to 150 Hu) [24, 25] (Fig. 1A, B, C). Aiming to standardize the approach and guarantee repeatability, the first slice including the upper plate of the 11th dorsal vertebra was considered as reference point to start the segmentation.

We considered the value < 30 Hu of the examined volume as threshold for the definition of muscle loss [15, 26, 27].

For the radiomics analysis, the radiomics plugin of 3D Slicer which encapsulates pyRadiomics library was applied. The previously segmented volume was loaded in the radiomics module interface. Range re-segmentation and intensity outlier filtering were not needed since the range for muscle assessment (− 29 to 150 Hu) was previously applied [28].

Aiming to avoid model overfitting due to the small sample size, only 72 radiomic features extracted (Fig. 1D). The radiomic features were of first and second order: intensity-based features (FOS) and texture features [gray-level co-occurrence matrix (GLCM) as well as gray-level run length matrix (GLRLM)]. Among the FOS, mean, *standard deviation*, *skewness*, *kurtosis*, and *energy* were included, while for the GLCM, for instance, *inverse variance*, *sum average*, *joint entropy,* and *cluster shade* were extracted; last, among the GLRLM, *short run emphasis*, *run percentage*, and *long run low gray level emphasis* were included.

Factor analysis was applied to select highly correlating radiomic features (Fig. 1E).

### Lung transplant allograft rejection

Allograft rejection was defined by a combination of tests and evaluations including: (i) immunological test, for instance demonstrating donor-specific circulating antigen; (ii) histopathological changes in the specimen obtained by transbronchial lung biopsy such as perivascular mononuclear cell infiltrates typical of acute rejection; (iii) visual assessment of HRCT scans, detecting findings such as ground-glass opacities and interstitial thickening in acute rejection; (iv) and pulmonary function test for example demonstrating 20% or greater decline in forced expiratory volume in the first second (FEV1) or forced vital capacity (FVC) from the best postoperative value in patients with chronic lung allograft disfunction [7, 29, 30]. In our tertiary center a weekly multidisciplinary meeting for monitoring lung transplanted patients and early detecting lung allograft rejection is performed.

### Statistical analysis

Descriptive statistics were performed. The Student’s *t*-test, the chi-square, and the Mann–Whitney tests were used, respectively, for continuous, categorical, and ordinal variables, to evaluate if any difference regarding muscle volume and the above-mentioned clinical and laboratory variables occurred between patients with and without muscle loss or between patients with and without allograft rejection.

To investigate the prognostic value of the features, previously selected by factor analysis, for allograft rejection, logistic regression analysis was used. In case of statistically significant results, the diagnostic accuracy was computed using receiver operating characteristic curves and the value with the highest Youden index was selected as cutoff.

To evaluate the robustness of the proposed method, all segmentations and data extraction were repeated by a second reader with four years of experience in chest imaging and the intraclass correlation coefficient (ICC) of the variables highly correlating at factor analysis, computed. ICC values > 0.750 were considered excellent [31].

All statistical analyses were performed with SPSS (IBM SPSS Statistics version 27, IBM Armonk, NY, USA), applying *p* < 0.05 as significant level.

## Results

**Fig. 2:**
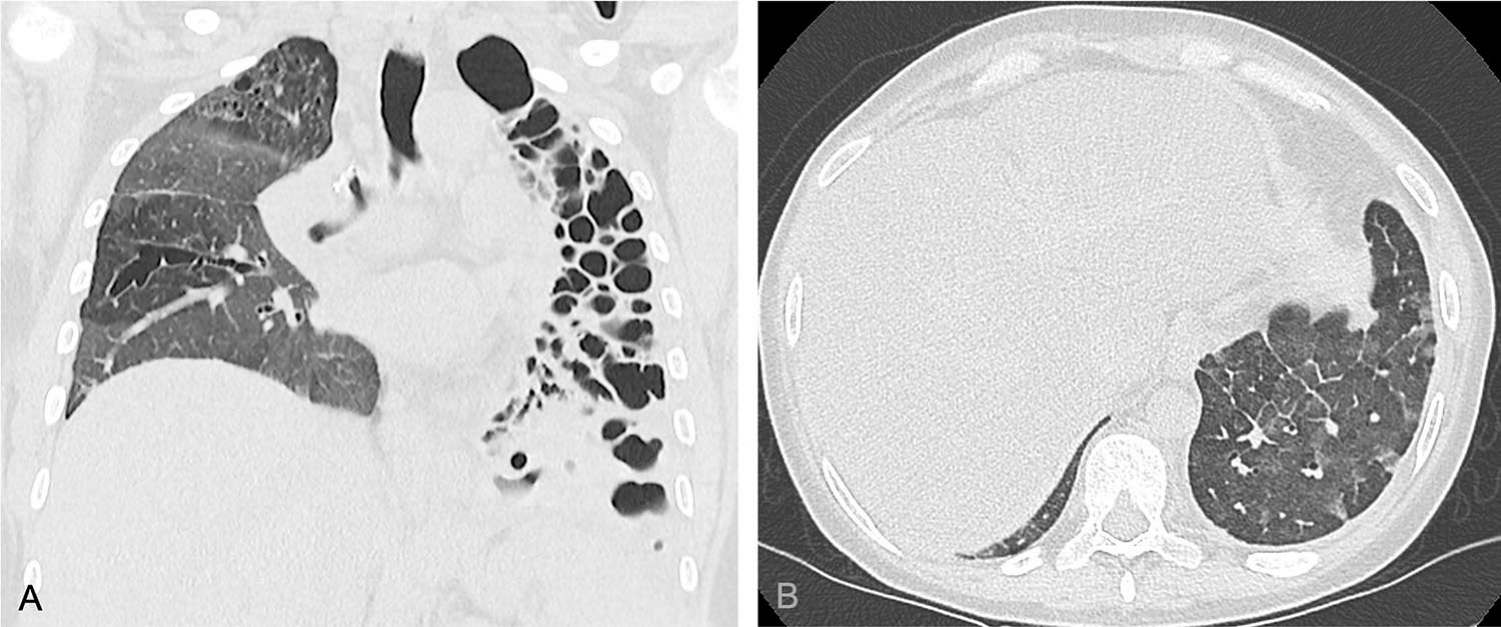
Forty-year-old female transplanted for cystic fibrosis who developed acute cellular allograft rejection six months after the transplant. In **A** the coronal reconstruction of the high resolution computed tomography performed before the bilateral lung transplant well demonstrating the severe bronchiectasis affecting the entire left lung as well as the bronchiectasis and ground-glass opacities in the right lung. In **B** an axial high resoution computed tomography image demonstrating ground-glass opacities and interlobular thickening in the left lower lobe suggesting acute allograft rejection then confirmed by laboratory and histological findings. The patient died a few weeks after the transplant

**Fig. 3:**
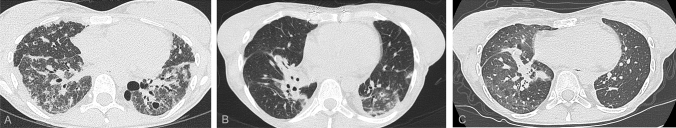
Twenty-four-year-old female transplanted for, histologically proven, lymphocytic interstitial pneumonia who developed restrictive chronic allograft rejection 20 months after the transplant. In **A** an axial image of the high-resolution computed tomography performed before the bilateral lung transplant well demonstrating bilateral severe interstitial thickening, bronchiectasis, and fibrotic ground-glass as well as cysts in the left lower lobe. In **B** an axial high-resolution computed tomography image demonstrating peri-hilar consolidations on the right side as well as band atelectasis and ground-glass opacities in the lower lobes; in **C** an axial image of the expiratory phase showing air trapping in the lower lobes. The diagnosis of restrictive chronic rejection was achieved including respiratory test as well as laboratory and histological findings

An overall number of 200 patients underwent a HRCT prior to the transplant. Nevertheless, only ninety-seven matched the inclusion criteria and were examined since 75 HRCT did not satisfy the technical criteria while 28 scans were performed earlier than two months before the transplant. The main characteristics of the examined population are summarized in Table 1. The investigated population was composed of 29 women (29.9%) and 68 men (70.1%) and the mean age was 50.4 years old. Most of the patients underwent bilateral lung transplant for idiopathic pulmonary fibrosis (*n* = 37, 38.1%), followed by cystic fibrosis (*n* = 12, 12.4%) (Table 1). Overall, 21 (21.6%) patients showed allograft rejection (i.e., fifteen acute and six chronic; Figs. 2, 3).

**Table 1:** Main characteristics of the examined population

Population characteristics		
Age (mean, SD)		50.4 (± 13) years old
Gender (frequency, percentage)		29 (30%) females 68 (70%) males
Pulmonary disease that led to the transplant (frequency, percentage)	Idiopathic pulmonary fibrosis	37 (38.1%)
	Cystic fibrosis	12 (12.4%)
	Chronic obstructive pulmonary disease	10 (10.3%)
	Sarcoidosis	4 (4.1%)
	Hypersensitivity pneumonia	4 (4.1%)
	Langherans cell histiocytosis	3 (3%)
	Lymphangioleyomiomatosis	3 (3%)
	Non-specific interstitial pneumonia	3 (3%)
	Others (including for instance one each of pulmonary hemosiderosis, primary pulmonary hypertension, lymphocytic interstitial pneumonia)	19 (19.6%)

The mean Hu value and volume of the examined spinal muscles were 36.6 ± 8 and 68.8 ± 54 cm^3^, respectively. Sixteen patients had radiological signs of muscle loss (i.e., Hu values < 30; in particular 23.2 ± 5 Hu), 13 were males and 10 affected by idiopathic pulmonary fibrosis (mean age 54.7 ± 9 years old). There were no differences of muscle volume neither between patients with and without muscle loss (70.8 ± 59 vs. 58.7 ± 12 cm^3^, *p* = 0.423) nor between patients developing or not allograft rejection (59 ± 16 vs. 71.7 ± 60 cm^3^, *p* = 0.334).

**Fig. 4:**
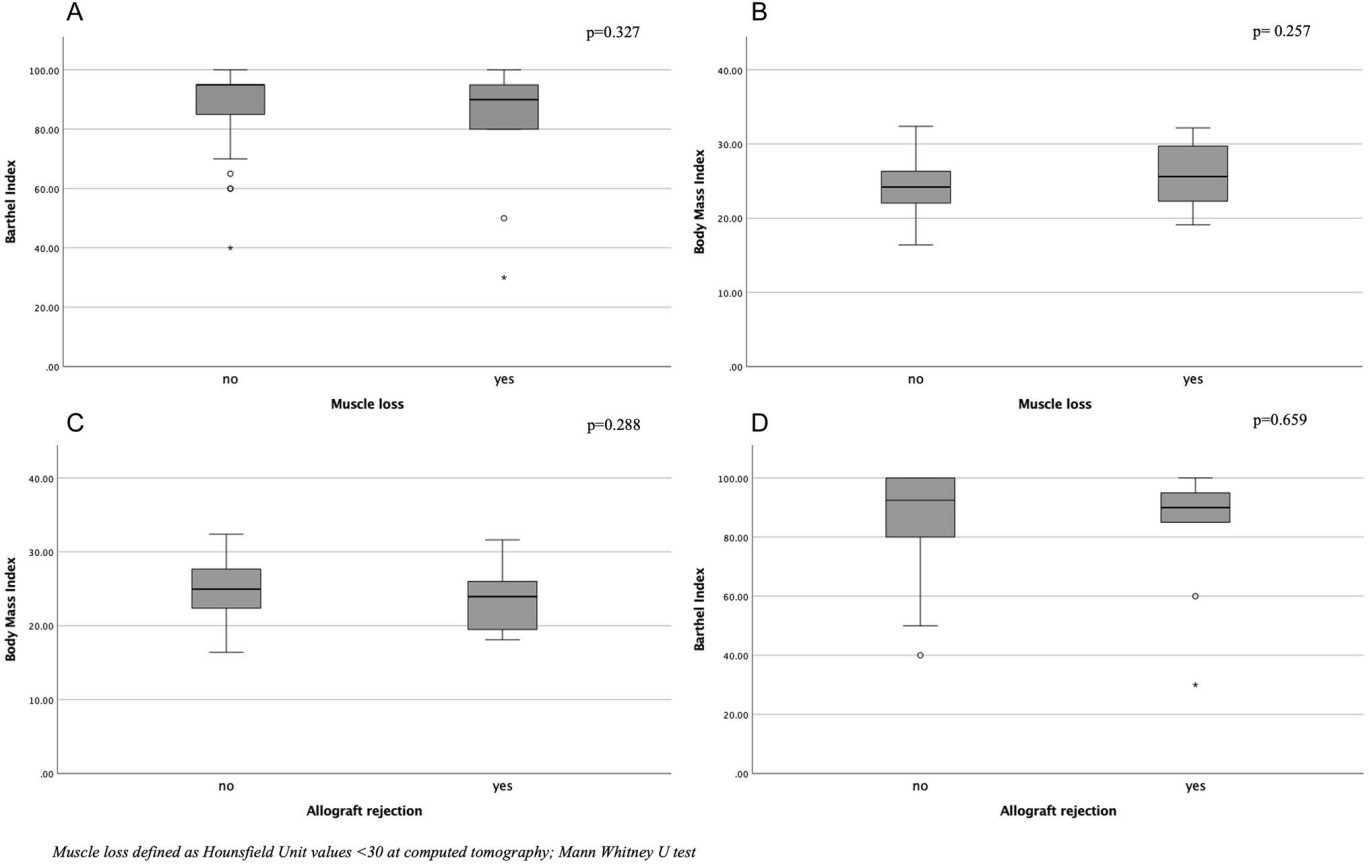
Box-plots demonstrating the lack of statistically significant differences regarding Barthel index and body mass index (BMI) in patients with or without muscles loss (**A** and **B**) and with or without allograft rejection (**C** and **D**), respectively

Patients with muscle loss showed significantly higher levels of inflammation expressed as ESR (43 ± 23 vs. 27.8 ± 19 mm/h, *p* = 0.023). Allograft rejection occurred most frequently in men (*p* = 0.016). None of the other clinical and laboratory variables, including BMI and Barthel index, showed any significant difference comparing patients with and without muscle loss as well as patients with and without allograft rejection (Table 2, Fig. 4).

**Table 2:** Comparisons of population characteristics according to muscle composition and allograft rejection

	Muscle loss*			Allograft rejection		
	Yes	No	*p*	Yes	No	*p*
Age (years old)	55 ± 9	50 ± 13	0.070	48 ± 13	51 ± 12	0.294
Gender (female/male)	4/14	25/54	0.572	11/10	18/58	**0.016**
Height (cm)	173.7 ± 6	170 ± 8.5	0.309	167 ± 9	172 ± 7	0.113
Weight (kg)	80 ± 13	71 ± 14	0.07	66 ± 15	74 ± 14	0.159
RBC (× 10^12^ L)	4.6 ± 0.5	4.7 ± 1	0.209	4.7 ± 1	4.7 ± 0.8	0.750
Hemoglobin (g/dl)	13.7 ± 1.7	14.1 ± 1.9	0.450	13.5 ± 2.5	14.1 ± 1.7	0.175
Hematocrit (vol%)	42 ± 5	42 ± 5	0.624	41 ± 7	43 ± 4	0.15
White blood cells (× 10^9^ L)	9 ± 3	9 ± 3	0.836	9 ± 5	9 ± 3	0.819
Creatinine (umol/L)	67 ± 15	73 ± 26	0.397	66.5 ± 22	73 ± 25	0.267
Albumin (g/dl)	3.8 ± 4	3.9 ± 5	0.467	3.9 ± 4	3.9 ± 5	0.814
ESR (mm/h)	43 ± 23	27.8 ± 19	**0.023**	30.8 ± 26	30.7 ± 19	0.996

Level of significance *p* < 0.05

Statistically significant results are given in bold

*RBC* red blood cell; *ESR* erythrocyte sedimentation rate

*patients with hounsfield unit muscle values < 30; ^#^Student’s *t*-test and chi-square test for continuous and categorical variables, respectively

**Fig. 5:**
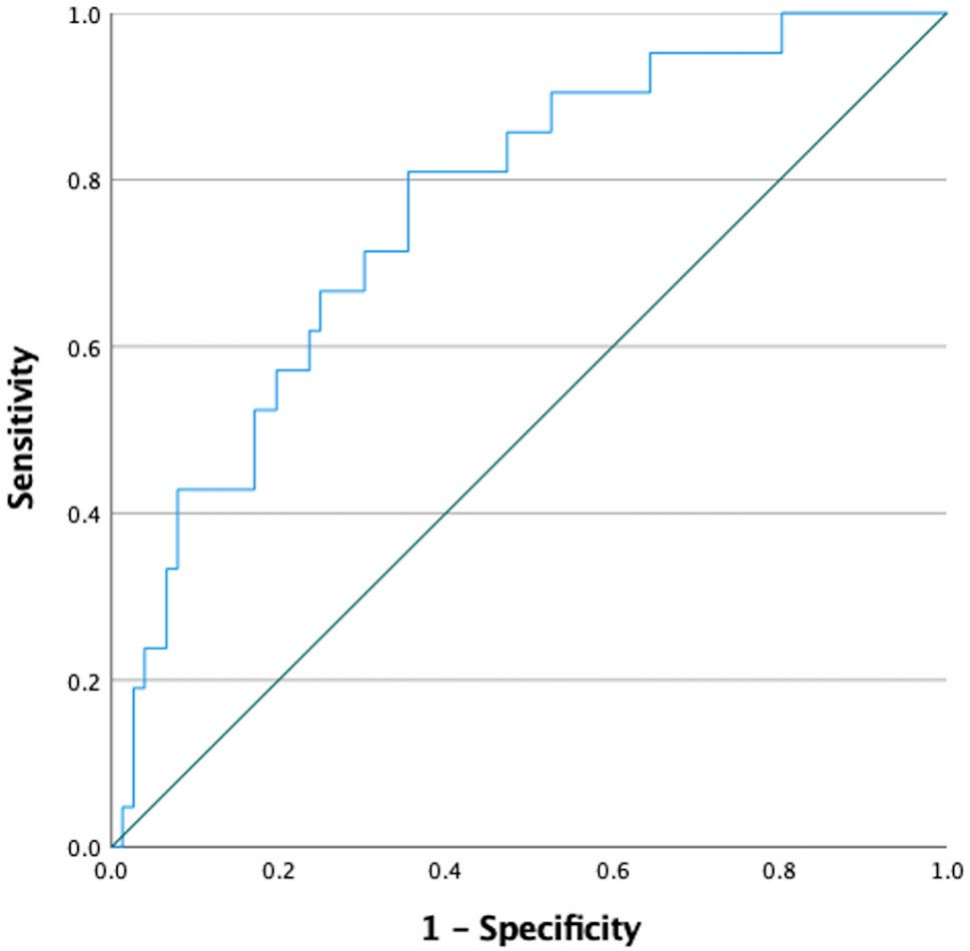
Receiver operating characteristic curve of the radiomic variable Imc2 extracted from the paravertebral muscle which demonstrated 76.6% accuracy in predicting allograft rejection

The following features were selected by factor analysis: *cluster prominence, Imc2, gray level non-uniformity normalized, median, kurtosis, gray level non-uniformity, and inverse variance*. The radiomics-based model including also Hu demonstrated that only the feature *Imc2* acts as a predictor of allograft rejection (*p* = 0.021). Such feature showed 76.6% accuracy and the value 0.19 demonstrated 81% sensitivity and 64.5% specificity in predicting lung transplant rejection (Fig. 5). The variables selected by factor analysis showed excellent repeatability: *cluster prominence* ICC = 0.984, 95%CI (0.976–0.989); *Imc2*, ICC = 0.970, 95% CI (0.956–0.980); *gray level non-uniformity normalized*, ICC = 0.955, 95% CI (0.934–0.970); *median*, ICC = 0.985, 95% CI (0.934–982); *kurtosis*, ICC = 0.937, 95% CI (0.907–0.957); *gray level non-uniformity,* ICC = 0.846, 95% CI (0.820–0.963); *inverse variance*, ICC = 0. 917, 95% CI (0.901–0.947).

## Discussion

Our study suggests that advanced CT analysis of paravertebral muscles may play a role in the prediction of allograft rejection in bilateral lung transplant candidates and that the radiomic variable *Imc2*, which quantifies the complexity of the texture in terms of correlation between the probability distributions of the elements of the matrix, could be then considered as a novel radiological biomarker [32]. Currently, the diagnosis of acute or chronic allograft rejection relies on a multidisciplinary approach including histopathology, radiology, functional and immunologic tests [4–7]. Diagnostic imaging is of support, for instance, leading to targeted transbronchial biopsy or, in case of suspected chronic allograft dysfunction, detecting signs compatible with bronchiolitis obliterans or restrictive allograft syndrome [7].

Despite our encouraging and novel results, it certainly has to be highlighted that the proposed model has moderate sensitivity and low specificity. Nevertheless, our accuracy is higher than the one reached by de Jong and colleagues (AUC 0.49), who used radiomics to predict muscle loss in patients affected by non-small cell lung cancer. Similarly, it is also higher than the one obtained by the model of Kim et al. when only the erector spinae (AUC range 0.55–0.75) was assessed in patients affected by the same type of tumor. In fact, this latter group of researchers has also demonstrated that a multifactorial model including gender, coarseness, skewness, and cluster prominence has a good performance in defining sarcopenia especially if all muscles of the thoracic wall at the level of the third lumbar vertebra are used (AUC > 0.75) [17, 18].

Although our results need to be further explored in multifactorial models, maybe expanding the analysis to all thoracic muscles, our evidence underlines the importance of a systemic approach to the overall management of lung transplant candidates. Indeed, the fact that muscle characteristics and strength may have an impact on the outcome is in agreement with previous studies showing that muscle composition plays a role on the general status and performance of patients after bilateral lung transplant [10–12]. Thus, our study further supports the evidence that several systemic factors may influence the success of the procedure [33–35].

It cannot be overlooked that the prognostic value of muscle loss assessed by CT is still controversial in the literature. In fact, Rozenberg et al. and Hsu and colleagues indicated that sarcopenia is a poor prognostic factor in patients undergoing bilateral lung transplant while our results are in accordance with Lee et al. who suggested that sarcopenia, assessed as muscle cross sectional area, does not affect the early outcome [9, 12, 36]. This heterogeneity of results could be due to the different muscle target used for the analysis (psoas vs. paraspinal muscles), to the type of assessment (single slice vs. volumetric), and to the extracted parameters (muscle density vs. muscle area).

The absence of differences in terms of allograft rejection between patients with and without muscle loss, lets us assume that *Imc2* may either reflect a very subtle onset of myosteatosis or more complex changes constituted of fat and inflammatory infiltrates considering also that significantly higher ESR occurred only in patients with muscle loss.

Certainly, correlating the radiological findings with muscle histological features could provide deeper insights into the characteristics of such changes. This type of investigation was not feasible in our study given the retrospective design of the project. Moreover, it cannot be overlooked that a biopsy of the paravertebral muscles would represent an invasive procedure. Considering the type of surgical intervention, a different muscle target, such as the diaphragm could be more easily accessible although it would be a challenge for the assessment at imaging. To overcome such difficulties, in future studies noninvasive techniques like diffusion tensor imaging, which enables the collection of quantitative information such as fractional anisotropy and mean diffusivity providing detailed information about muscle structure, could be compared or even associated with radiomics to provide new insights into the changes occurring in the paravertebral muscles of this group of patients [37, 38].

This study is affected by several limits. First, we did not assess the prognostic value of muscle composition on the overall outcome because this information was not available for most of the patients. Certainly, further studies including also this type of analysis are needed to further investigate the role of radiomics in transplantology.

We did not subdivide our patients according to the type of rejection (i.e., acute or chronic) because of the overall low number of cases of allograft dysfunction. It has to be underlined that our rate of allograft rejection is in the range reported in the literature and that this limit could be overcome by multicenter studies with a larger sample size [5].

Given the retrospective study design, we could not investigate the role of additional clinical and laboratory variables, such as the levels of creatine phosphokinase because they were not available for all or at least most of the included patients. Prospective studies with tailored collections of muscle-related laboratory and clinical variables are highly recommended. Moreover, further muscle parameters like the skeletal muscle index and/or the application of additional methods like DXA to assess muscle loss were not used but our study was focused on a CT-based radiomic evaluation of a muscle volume extracted from the paravertebral area [14, 39]. In future a multiparameter model including also these elements may be developed. Last, we must address that from a pool of 200 patients only 97 were included. This is given to the retrospective study design with a large time interval. In fact, given the technical improvements of the last decade, most of the excluded HRCT scans were performed with different techniques and heterogeneous parameters. Certainly, future prospective studies may overcome all limits. Nevertheless, considering that bilateral lung transplant is performed for rare diseases, we consider the size of our population satisfying for this preliminary study.

In conclusion, in our population the use of a muscle CT-based model of radiomics allowed the prediction of allograft dysfunction in lung transplanted patients. This evidence, if confirmed in larger, multicentric, and prospective studies, may improve the overall clinical management of patients undergoing bilateral lung transplant, influence the selection of optimal candidates, and guide the development of specific tailored physiotherapy programs in patients considered at risk at imaging.

## References

[1] Adegunsoye A, Strek ME, Garrity E, Guzy R, Bag R (2017). Comprehensive care of the lung transplant patient. Chest.

[2] Venuta F, Van Raemdonck D (2017). History of lung transplantation. J Thorac Dis.

[3] Sun H, Deng M, Chen W, Liu M, Dai H, Wang C (2022). Graft dysfunction and rejection of lung transplant, a review on diagnosis and management. Clin Respir J.

[4] Verleden GM, Glanville AR, Lease ED, Fisher AJ, Calabrese F, Corris AP (2019). Chronic lung allograft dysfunction: definition, diagnostic criteria, and approaches to treatment: a consensus report from the pulmonary council of the ISHLT. J Heart Lung Transplant.

[5] Parulekar AD, Kao CC (2019). Detection, classification, and management of rejection after lung transplantation. J Thorac Dis.

[6] Berry G, Burke M, Andersen C, Angelini A, Bruneval P, Calabrese F (2013). Pathology of pulmonary antibody-mediated rejection: 2012 update from the pathology council of the ISHLT. J Heart Lung Transplant.

[7] Bin Saeedan M, Mukhopadhyay S, Lane CR, Renapurkar RD (2020). Imaging indications and findings in evaluation of lung transplant graft dysfunction and rejection. Insights Imaging.

[8] Hota P, Dass C, Kumaran M, Simpson S (2018). High-resolution CT findings of obstructive and restrictive phenotypes of chronic lung allograft dysfunction: more than just bronchiolitis obliterans syndrome. Am J Roentgenol.

[9] Lee S, Paik HC, Haam SJ, Lee CY, Nam KS, Jung HS (2016). Sarcopenia of thoracic muscle mass is not a risk factor for survival in lung transplant recipients. J Thorac Dis.

[10] Langer D, Burtin C, Schepers L, Ivanova A, Verleden G, Decramer M (2012). Exercise training after lung transplantation improves participation in daily activity: a randomized controlled trial. Am J Transplant.

[11] Vivodtzev I, Pison C, Guerrero K, Mezin P, Maclet E, Borel JC (2011). Benefits of home- based endurance training in lung transplant recipients. Respir Physiol Neurobiol.

[12] Hsu J, Krishnan A, Lin CT, Shah PD, Broderick SR, Higgins RSD (2019). Sarcopenia of the psoas muscles is associated with poor outcomes following lung transplantation. Ann Thorac Surg.

[13] Stringer HJ, Wilson D (2018). The role of ultrasound as a diagnostic tool for sarcopenia. J Frailty Aging.

[14] Messina C, Maffi G, Vitale JA, Ulivieri FM, Guglielmi G, Sconfienza LM (2018). Diagnostic imaging of osteoporosis and sarcopenia: a narrative review. Quant Imaging Med Surg.

[15] Giraudo C, Cavaliere AC, Lupi A, Guglielmi G, Quaia E (2020). Established paths and new avenues: a review of the main radiological techniques for investigating sarcopenia. Quant Imaging Med Surg.

[16] Arayne AA, Gartrell R, Qiao J, Baird PN, Yeung JM (2023). Comparison of CT derived body composition at the thoracic T4 and T12 with lumbar L3 vertebral levels and their utility in patients with rectal cancer. BMC Cancer.

[17] Kim YJ (2021). Machine learning models for sarcopenia identification based on radiomic features of muscles in computed tomography. Int J Environ Res Public Health.

[18] de Jong EEC, Sander KJC, Deist TM, van Elmpt W, Jochems A, van Timmeren JE (2019). Can radiomics help to predict skeletal muscle response to chemotherapy in stage IV non-small cell lung cancer?. Eur J Cancer.

[19] Dong X, Dan X, Yawen A, Haibo X, Huan L, Mengqi T (2020). Identifying sarcopenia in advanced non-small cell lung cancer patients using skeletal muscle CT radiomics and machine learning. Thorac Cancer.

[20] Gillies RJ, Kinahan PE, Kricak H (2016). Radiomics: Images are more than pictures, they are data. Radiology.

[21] Shur JD, Doran SJ, Kumar S, Ap Dafydd D, Downey K, O’Connor JPB (2021). Radiomics in oncology: a practical guide. Radiographics.

[22] Giraudo C, Frattin G, Fichera G, Motta R, Stramare R (2021). A practical integrated radiomics model predicting intensive care hospitalization in COVID-19. Crit Care.

[23] Chen Y, Li H, Feng J, Suo S, Feng Q, Shen J (2021). A novel radiomics nomogram for the prediction of secondary loss of response to infliximab in crohn’s disease. J Inflamm Res.

[24] Amini B, Boyle SP, Boutin RD, Lechnik L (2019). Approaches to assessment of muscle mass and myosteatosis on computed tomography: a systematic review. J Gerontol A Biol Sci Med Sci.

[25] Rollins KE, Gopinath A, Awwad A, MacDonald IA, Lobo DN (2020). Computed tomography-based psoas skeletal muscle area and radiodensity are poor sentinels for whole L3 skeletal muscle values. Clin Nutr.

[26] Giraudo C, Librizzi G, Fichera G, Motta R, Balestro E, Calabrese F (2021). Reduced muscle mass as predictor of intensive care unit hospitalization in COVID-19 patients. PLoS ONE.

[27] Aubrey J, Esfandiari N, Baracos VE, Buteau FA, Frenette J, Putman CT (2014). Measurement of skeletal muscle radiation attenuation and basis of its biological variation. Acta Physiol (Oxf).

[28] van Timmeren JE, Cester D, Tanadini-Lang S, Alkadhi H, Baessler B (2020). Radiomics in medical imaging-"how-to" guide and critical reflection. Insights Imaging.

[29] Roden AC, Tazelaar HD (2018). Pathology of lung rejection: cellular and humoral mediated. Lung Transplant.

[30] Kotecha S, Paraskeva MA, Levin K, Snell GI (2020). An update on chronic lung allograft dysfunction. Ann Transl Med.

[31] Cicchetti DV (1994). Guidelines, criteria, and rules of thumb for evaluating normed and standardized assessment instruments in psychology. Psychol Assess.

[32] Zwanenburg A, Vallieres M, Abdalah MA, Aerts HJW, Andrearczyk V, Apte A (2020). The Image biomarker standardization initiative: standardized quantitative radiomics for high-throughput image-based phenotyping. Radiology.

[33] Venado A, Kolaitis NA, Huang CY, Gao Y, Glidden DV, Soong A (2020). Frailty after lung transplantation is associated with impaired health-related quality of life and mortality. Thorax.

[34] Weill D (2018). Lung transplantation: indications and contraindications. J Thorac Dis.

[35] Hook JL, Lederer DJ (2012). Selecting lung transplant candidates: Where do current guidelines fall short?. Expert Rev Respir Med.

[36] Rozenberg D, Wickerson L, Singer LG, Mathur S (2014). Sarcopenia in lung transplantation: a systematic review. J Heart Lung Transplant.

[37] Giraudo C, Motyka S, Weber M, Feiweier T, Trattnig S, Bogner W (2019). Diffusion tensor imaging of healthy skeletal muscles: a comparison between 7 T and 3 T. Investig Radiol.

[38] Giraudo C, Motyka S, Weber M, Karner M, Resinger C, Feiweier T (2018). Normalized STEAM-based diffusion tensor imaging provides a robust assessment of muscle tears in football players: preliminary results of a new approach to evaluate muscle injuries. Eur Radiol.

[39] Prado CMM, Lieffers JR, McCargar LJ, Reiman T, Sawyer MB, Martin L (2008). Prevalence and clinical implications of sarcopenic obesity in patients with solid tumours of the respiratory and gastrointestinal tracts: a population-based study. Lancet Oncol.

